# Supplementary material for: Effectiveness of Rhizophagus intraradices and Acinetobacter calcoaceticus on soybean growth and thiram residues in soybean grains and rhizosphere soil
Source: PeerJ. 2025 Jul 11;13:e19701. doi: 10.7717/peerj.19701 (PMC12258158; doi:10.7717/peerj.19701)
Supplement: Supplemental Information 1 — Blank control (CK); inoculation with R. intraradices (R); inoculation with A. calcoaceticus (A); inoculation with R. intraradices and A. calcoaceticus (RA); thiram spraying only (TK); thiram spraying and inoculation with R. intraradices (TR); thiram spraying and inoculation with A. calcoaceticus (TA); and thiram spraying and inoculation with R. intraradices and A. calcoaceticus (TRA). Values are means ±standard error with three replicates. Analysis of variance (ANOVA) and Duncan’s test (honestly significant difference, HSD) were applied to evaluate significant differences (P < 0.05). Different lowercase letters indicate significant differences from different treatments (P < 0.05). [file peerj-13-19701-s001.docx]

**Table 1-1. Effects of treatments on the AMF spore density.**

| **Treatments** | Date-1 | Date-2 | Date-3 | the means ± the standard error |
| --- | --- | --- | --- | --- |
| R | 2.25 | 2.49 | 2.29 | 2.34±0.13^b^ |
| A | 1.18 | 1.31 | 1.6 | 1.36±0.22d |
| RA | 4.23 | 3.93 | 3.77 | 3.98±0.23a |
| CK | 0.77 | 0.68 | 0.75 | 0.73±0.05f |
| TR | 1.12 | 1.08 | 1.05 | 1.08±0.04e |
| TA | 0.37 | 0.46 | 0.46 | 0.43±0.05g |
| TRA | 1.72 | 1.67 | 1.51 | 1.63±0.11c |
| TK | 0.23 | 0.19 | 0.26 | 0.23±0.04g |

Note: Blank control (CK); inoculation with *R. intraradices* (R); inoculation with *A. calcoaceticus* (A); inoculation with *R. intraradices* and *A. calcoaceticus* (RA); thiram spraying only (TK); thiram spraying and inoculation with *R. intraradices* (TR); thiram spraying and inoculation with *A. calcoaceticus* (TA); and thiram spraying and inoculation with *R. intraradices* and *A. calcoaceticus* (TRA). Values are means ± standard error with three replicates. Analysis of variance (ANOVA) and Duncan’s test (honestly significant difference, HSD) were applied to evaluate significant differences (*P* < 0.05). Different lowercase letters indicate significant differences from different treatments (*P* < 0.05).

**Table 1-2. Effects of treatments on the AMF infection rate.**

| **Treatments** | Date-1 | Date-2 | Date-3 | the means ± the standard error |
| --- | --- | --- | --- | --- |
| R | 91.37 | 89.25 | 92.08 | 90.90±0.85b |
| A | 60.45 | 58.82 | 61.70 | 60.32±0.83e |
| RA | 96.39 | 95.20 | 97.58 | 96.39±0.69a |
| CK | 47.84 | 45.69 | 49.03 | 47.52±0.98g |
| TR | 82.71 | 83.62 | 80.98 | 82.44±0.77d |
| TA | 51.78 | 52.57 | 51.93 | 52.09±0.24f |
| TRA | 84.65 | 86.02 | 85.31 | 85.33±0.40c |
| TK | 44.29 | 43.05 | 42.82 | 43.39±0.46h |

Note: Blank control (CK); inoculation with *R. intraradices* (R); inoculation with *A. calcoaceticus* (A); inoculation with *R. intraradices* and *A. calcoaceticus* (RA); thiram spraying only (TK); thiram spraying and inoculation with *R. intraradices* (TR); thiram spraying and inoculation with *A. calcoaceticus* (TA); and thiram spraying and inoculation with *R. intraradices* and *A. calcoaceticus* (TRA). Values are means ± standard error with three replicates. Analysis of variance (ANOVA) and Duncan’s test (honestly significant difference, HSD) were applied to evaluate significant differences (*P* < 0.05). Different lowercase letters indicate significant differences from different treatments (*P* < 0.05).

**Table 1-3. Effects of treatments on the incidence of soybean root rot.**

| **Treatments** | Date-1 | Date-2 | Date-3 | the means ± the standard error |
| --- | --- | --- | --- | --- |
| R | 0.30 | 0.35 | 0.30 | 31.67±0.03^de^ |
| A | 0.40 | 0.40 | 0.35 | 38.33±0.03^cde^ |
| RA | 0.20 | 0.20 | 0.20 | 20.00±0.00^f^ |
| CK | 0.80 | 0.75 | 0.70 | 75.00±0.05^a^ |
| TR | 0.40 | 0.35 | 0.45 | 40.00±0.05^cd^ |
| TA | 0.40 | 0.45 | 0.40 | 41.67±0.03^bc^ |
| TRA | 0.35 | 0.25 | 0.30 | 30.00±0.05^e^ |
| TK | 0.60 | 0.50 | 0.40 | 50.00±0.10^b^ |

Note: Blank control (CK); inoculation with *R. intraradices* (R); inoculation with *A. calcoaceticus* (A); inoculation with *R. intraradices* and *A. calcoaceticus* (RA); thiram spraying only (TK); thiram spraying and inoculation with *R. intraradices* (TR); thiram spraying and inoculation with *A. calcoaceticus* (TA); and thiram spraying and inoculation with *R. intraradices* and *A. calcoaceticus* (TRA). Values are means ± standard error with three replicates. Analysis of variance (ANOVA) and Duncan’s test (honestly significant difference, HSD) were applied to evaluate significant differences (*P* < 0.05). Different lowercase letters indicate significant differences from different treatments (*P* < 0.05).

**Table 1-4. Effects of treatments on the nodule number.**

| **Treatments** | Date-1 | Date-2 | Date-3 | the means ± the standard error |
| --- | --- | --- | --- | --- |
| R | 71 | 68 | 73 | 70.67±2.52^c^ |
| A | 65 | 61 | 60 | 62.00±2.65^d^ |
| RA | 112 | 108 | 107 | 109.00±2.65^a^ |
| CK | 29 | 37 | 31 | 32.33±4.16^f^ |
| TR | 52 | 49 | 53 | 51.33±2.08^e^ |
| TA | 45 | 41 | 37 | 41.00±4.00^f^ |
| TRA | 83 | 86 | 79 | 82.67±3.51^b^ |
| TK | 31 | 35 | 33 | 33.00±2.00^f^ |

Note: Blank control (CK); inoculation with *R. intraradices* (R); inoculation with *A. calcoaceticus* (A); inoculation with *R. intraradices* and *A. calcoaceticus* (RA); thiram spraying only (TK); thiram spraying and inoculation with *R. intraradices* (TR); thiram spraying and inoculation with *A. calcoaceticus* (TA); and thiram spraying and inoculation with *R. intraradices* and *A. calcoaceticus* (TRA). Values are means ± standard error with three replicates. Analysis of variance (ANOVA) and Duncan’s test (honestly significant difference, HSD) were applied to evaluate significant differences (*P* < 0.05). Different lowercase letters indicate significant differences from different treatments (*P* < 0.05).

**Table 1-5. Effects of treatments on total number of bacterial colonies in the rhizosphere soil of soybean plants.**

| **Treatments** | Date-1 | Date-2 | Date-3 | the means ± the standard error |
| --- | --- | --- | --- | --- |
| R | 11.12×10^5^ | 12.35×10^5^ | 10.86×10^5^ | (1.14±0.80)×10^6b^ |
| A | 9.03×10^5^ | 9.23×10^5^ | 8.88×10^5^ | (9.05±0.18)×10^5c^ |
| RA | 16.56×10^5^ | 16.68×10^5^ | 16.49×10^5^ | (1.66±0.10)×10^6a^ |
| CK | 7.20×10^5^ | 7.12×10^5^ | 7.05×10^5^ | (7.12±0.08)×10^5e^ |
| TR | 6.67×10^5^ | 6.76×10^5^ | 6.51×10^5^ | (6.65±0.13)×10^5e^ |
| TA | 5.87×10^5^ | 6.02×10^5^ | 5.73×10^5^ | (5.87±0.15)×10^5f^ |
| TRA | 8.52×10^5^ | 8.35×10^5^ | 8.61×10^5^ | (8.49±0.13)×10^5d^ |
| TK | 3.69×10^5^ | 3.72×10^5^ | 3.60×10^5^ | (3.67±0.06)×10^5g^ |

Note: Blank control (CK); inoculation with *R. intraradices* (R); inoculation with *A. calcoaceticus* (A); inoculation with *R. intraradices* and *A. calcoaceticus* (RA); thiram spraying only (TK); thiram spraying and inoculation with *R. intraradices* (TR); thiram spraying and inoculation with *A. calcoaceticus* (TA); and thiram spraying and inoculation with *R. intraradices* and *A. calcoaceticus* (TRA). Values are means ± standard error with three replicates. Analysis of variance (ANOVA) and Duncan’s test (honestly significant difference, HSD) were applied to evaluate significant differences (*P* < 0.05). Different lowercase letters indicate significant differences from different treatments (*P* < 0.05).

**Table 2-1. Effects of treatments on soybean biomass (Plant Heigh).**

| **Treatments** | Date-1 | Date-2 | Date-3 | the means ± the standard error |
| --- | --- | --- | --- | --- |
| R | 59.12 | 62.44 | 61.15 | 60.90±1.67^b^ |
| A | 59.21 | 57.81 | 58.87 | 58.63±0.73^c^ |
| RA | 64.98 | 67.91 | 65.32 | 66.07±1.60^a^ |
| CK | 56.3 | 55.51 | 55.09 | 55.63±0.61^e^ |
| TR | 59.72 | 56. 11 | 58.38 | 58.07±1.82^cd^ |
| TA | 55.31 | 56.61 | 56. 18 | 56.03±0.66^de^ |
| TRA | 60.19 | 60.62 | 62. 49 | 61.10±1.22^b^ |
| TK | 56.4 | 56.87 | 56. 14 | 56.47±0.37^cde^ |

Note: Blank control (CK); inoculation with *R. intraradices* (R); inoculation with *A. calcoaceticus* (A); inoculation with *R. intraradices* and *A. calcoaceticus* (RA); thiram spraying only (TK); thiram spraying and inoculation with *R. intraradices* (TR); thiram spraying and inoculation with *A. calcoaceticus* (TA); and thiram spraying and inoculation with *R. intraradices* and *A. calcoaceticus* (TRA). Values are means ± standard error with three replicates. Analysis of variance (ANOVA) and Duncan’s test (honestly significant difference, HSD) were applied to evaluate significant differences (*P* < 0.05). Different lowercase letters indicate significant differences from different treatments (*P* < 0.05).

**Table 2-2. Effects of treatments on soybean biomass (Stem Diameter).**

| **Treatments** | Date-1 | Date-2 | Date-3 | the means ± the standard error |
| --- | --- | --- | --- | --- |
| R | 6.41 | 6.11 | 6.50 | 6.34±0.20^b^ |
| A | 6.08 | 6.28 | 6.41 | 6.26±0.16^b^ |
| RA | 6.84 | 6.72 | 6.85 | 6.80±0.07^a^ |
| CK | 5.83 | 5.26 | 5.47 | 5.52±0.28^d^ |
| TR | 5.85 | 5.98 | 6.29 | 6.04±0.22^bc^ |
| TA | 6.28 | 5.91 | 5.34 | 5.84±0.47^bcd^ |
| TRA | 6.35 | 6.18 | 6.37 | 6.30±0.10^b^ |
| TK | 5.74 | 5.37 | 6.02 | 5.71±0.32^cd^ |

Note: Blank control (CK); inoculation with *R. intraradices* (R); inoculation with *A. calcoaceticus* (A); inoculation with *R. intraradices* and *A. calcoaceticus* (RA); thiram spraying only (TK); thiram spraying and inoculation with *R. intraradices* (TR); thiram spraying and inoculation with *A. calcoaceticus* (TA); and thiram spraying and inoculation with *R. intraradices* and *A. calcoaceticus* (TRA). Values are means ± standard error with three replicates. Analysis of variance (ANOVA) and Duncan’s test (honestly significant difference, HSD) were applied to evaluate significant differences (*P* < 0.05). Different lowercase letters indicate significant differences from different treatments (*P* < 0.05).

**Table 2-3. Effects of treatments on soybean biomass (Root Length).**

| **Treatments** | Date-1 | Date-2 | Date-3 | the means ± the standard error |
| --- | --- | --- | --- | --- |
| R | 24.79 | 25.73 | 26.06 | 25.53±0.66^c^ |
| A | 23.89 | 23.54 | 24.47 | 23.97±0.47^d^ |
| RA | 30.73 | 29.25 | 30.54 | 30.17±0.81^a^ |
| CK | 19.19 | 20.27 | 20.62 | 20.03±0.75^e^ |
| TR | 22.34 | 23.86 | 24.38 | 23.53±1.06^d^ |
| TA | 22.37 | 22.38 | 24.03 | 22.93±0.96^d^ |
| TRA | 28.01 | 27.67 | 27.86 | 28.00±0.17^b^ |
| TK | 18.63 | 18.25 | 18.72 | 18.53±0.25^f^ |

Note: Blank control (CK); inoculation with *R. intraradices* (R); inoculation with *A. calcoaceticus* (A); inoculation with *R. intraradices* and *A. calcoaceticus* (RA); thiram spraying only (TK); thiram spraying and inoculation with *R. intraradices* (TR); thiram spraying and inoculation with *A. calcoaceticus* (TA); and thiram spraying and inoculation with *R. intraradices* and *A. calcoaceticus* (TRA). Values are means ± standard error with three replicates. Analysis of variance (ANOVA) and Duncan’s test (honestly significant difference, HSD) were applied to evaluate significant differences (*P* < 0.05). Different lowercase letters indicate significant differences from different treatments (*P* < 0.05).

**Table 2-4. Effects of treatments on soybean biomass (Fresh Weight).**

| **Treatments** | Date-1 | Date-2 | Date-3 | the means ± the standard error |
| --- | --- | --- | --- | --- |
| R | 65.2 | 63.8 | 67.9 | 65.63±2.08^bc^ |
| A | 60.3 | 64.7 | 57.9 | 60.97±3.44^c^ |
| RA | 70.9 | 75.7 | 72.1 | 72.90±2.49^a^ |
| CK | 49.2 | 42.9 | 45.1 | 45.73±3.19^e^ |
| TR | 58.1 | 52.3 | 55.2 | 55.20±2.9^d^ |
| TA | 50.2 | 55.3 | 48.3 | 51.27±3.61^d^ |
| TRA | 67.7 | 68.3 | 67.5 | 67.83±0.41^b^ |
| TK | 44.3 | 45.2 | 40.3 | 43.27±2.60^e^ |

Note: Blank control (CK); inoculation with *R. intraradices* (R); inoculation with *A. calcoaceticus* (A); inoculation with *R. intraradices* and *A. calcoaceticus* (RA); thiram spraying only (TK); thiram spraying and inoculation with *R. intraradices* (TR); thiram spraying and inoculation with *A. calcoaceticus* (TA); and thiram spraying and inoculation with *R. intraradices* and *A. calcoaceticus* (TRA). Values are means ± standard error with three replicates. Analysis of variance (ANOVA) and Duncan’s test (honestly significant difference, HSD) were applied to evaluate significant differences (*P* < 0.05). Different lowercase letters indicate significant differences from different treatments (*P* < 0.05).

**Table 2-5. Effects of treatments on soybean biomass (Aboveground Dry Weigh).**

| **Treatments** | Date-1 | Date-2 | Date-3 | the means ± the standard error |
| --- | --- | --- | --- | --- |
| R | 26.5 | 25.2 | 24.5 | 25.40±1.01^c^ |
| A | 22.8 | 25.6 | 25.1 | 24.50±1.49^cd^ |
| RA | 32.1 | 31.5 | 30.3 | 31.30±0.91^a^ |
| CK | 18.7 | 19.2 | 17.2 | 18.37±1.04^f^ |
| TR | 23.1 | 22.1 | 22.8 | 22.67±0.51^de^ |
| TA | 22.6 | 20.2 | 23.2 | 22.00±1.58^e^ |
| TRA | 27.6 | 28.2 | 27.1 | 27.63±0.55^b^ |
| TK | 17.1 | 15.2 | 14.1 | 15.47±1.51^g^ |

Note: Blank control (CK); inoculation with *R. intraradices* (R); inoculation with *A. calcoaceticus* (A); inoculation with *R. intraradices* and *A. calcoaceticus* (RA); thiram spraying only (TK); thiram spraying and inoculation with *R. intraradices* (TR); thiram spraying and inoculation with *A. calcoaceticus* (TA); and thiram spraying and inoculation with *R. intraradices* and *A. calcoaceticus* (TRA). Values are means ± standard error with three replicates. Analysis of variance (ANOVA) and Duncan’s test (honestly significant difference, HSD) were applied to evaluate significant differences (*P* < 0.05). Different lowercase letters indicate significant differences from different treatments (*P* < 0.05).

**Table 2-6. Effects of treatments on soybean biomass (Underground Dry Weigh).**

| **Treatments** | Date-1 | Date-2 | Date-3 | the means ± the standard error |
| --- | --- | --- | --- | --- |
| R | 3.5 | 3.3 | 3.6 | 3.47±0.15^bc^ |
| A | 3.6 | 3.3 | 2.6 | 3.17±0.51^c^ |
| RA | 4.2 | 4.3 | 4.7 | 4.4±0.26^a^ |
| CK | 2.6 | 2.4 | 2.2 | 2.40±0.20^d^ |
| TR | 3.3 | 3.0 | 3.4 | 3.23±0.20^c^ |
| TA | 3.3 | 3.1 | 3.0 | 3.13±0.15^c^ |
| TRA | 3.8 | 4.0 | 3.9 | 3.90±0.10^b^ |
| TK | 2.6 | 2.4 | 2.1 | 2.37±0.25^d^ |

Note: Blank control (CK); inoculation with *R. intraradices* (R); inoculation with *A. calcoaceticus* (A); inoculation with *R. intraradices* and *A. calcoaceticus* (RA); thiram spraying only (TK); thiram spraying and inoculation with *R. intraradices* (TR); thiram spraying and inoculation with *A. calcoaceticus* (TA); and thiram spraying and inoculation with *R. intraradices* and *A. calcoaceticus* (TRA). Values are means ± standard error with three replicates. Analysis of variance (ANOVA) and Duncan’s test (honestly significant difference, HSD) were applied to evaluate significant differences (*P* < 0.05). Different lowercase letters indicate significant differences from different treatments (*P* < 0.05).

**Table 2-7. Effects of treatments on soybean biomass (Yield per Plant).**

| **Treatments** | Date-1 | Date-2 | Date-3 | the means ± the standard error |
| --- | --- | --- | --- | --- |
| R | 23.55 | 23.69 | 23.82 | 23.69±0.14^b^ |
| A | 22.03 | 22.14 | 21.83 | 22.00±0.16^c^ |
| RA | 27.06 | 27.18 | 26.68 | 26.97±0.26^a^ |
| CK | 19.19 | 19.68 | 19.95 | 19.61±0.39^e^ |
| TR | 20.97 | 21.59 | 23.00 | 21.9±1.04^cd^ |
| TA | 19.74 | 20.13 | 20.30 | 20.06±0.29^e^ |
| TRA | 23.55 | 23.48 | 23.88 | 23.64±0.21^b^ |
| TK | 21.14 | 21.34 | 21.14 | 21.21±0.12^d^ |

Note: Blank control (CK); inoculation with *R. intraradices* (R); inoculation with *A. calcoaceticus* (A); inoculation with *R. intraradices* and *A. calcoaceticus* (RA); thiram spraying only (TK); thiram spraying and inoculation with *R. intraradices* (TR); thiram spraying and inoculation with *A. calcoaceticus* (TA); and thiram spraying and inoculation with *R. intraradices* and *A. calcoaceticus* (TRA). Values are means ± standard error with three replicates. Analysis of variance (ANOVA) and Duncan’s test (honestly significant difference, HSD) were applied to evaluate significant differences (*P* < 0.05). Different lowercase letters indicate significant differences from different treatments (*P* < 0.05).

**Table 3-1. Effects of treatments on thiram residues in soybean grains.**

| **Treatments** | Date-1 | Date-2 | Date-3 | the means ± the standard error |
| --- | --- | --- | --- | --- |
| CK | 0.00 | 0.00 | 0.00 | 0.00±0.00^e^ |
| TK | 7.37 | 7.40 | 7.36 | 7.38±0.02^a^ |
| TR | 3.56 | 3.60 | 3.51 | 3.56±0.05^c^ |
| TA | 4.59 | 4.58 | 4.59 | 4.59±0.01^b^ |
| TRA | 2.01 | 2.01 | 1.94 | 1.99±0.04^d^ |

Note: Blank control (CK); thiram spraying only (TK); thiram spraying and inoculation with *R. intraradices* (TR); thiram spraying and inoculation with *A. calcoaceticus* (TA); and thiram spraying and inoculation with *R. intraradices* and *A. calcoaceticus* (TRA). Values are means ± standard error with three replicates. Analysis of variance (ANOVA) and Duncan’s test (honestly significant difference, HSD) were applied to evaluate significant differences (*P* < 0.05). Different lowercase letters indicate significant differences from different treatments (*P* < 0.05).

**Table 3-2. Effects of treatments on thiram residues in soybean rhizosphere soil.**

| **Treatments** | Date-1 | Date-2 | Date-3 | the means ± the standard error |
| --- | --- | --- | --- | --- |
| CK | 0.00 | 0.00 | 0.00 | 0.00±0.00^e^ |
| TK | 7.27 | 7.28 | 7.25 | 7.27±0.02^a^ |
| TR | 4.48 | 4.48 | 4.43 | 4.46±0.03^b^ |
| TA | 3.85 | 3.82 | 3.79 | 3.82±0.03^c^ |
| TRA | 2.26 | 2.29 | 2.22 | 2.26±0.02^d^ |

Note: Blank control (CK); thiram spraying only (TK); thiram spraying and inoculation with *R. intraradices* (TR); thiram spraying and inoculation with *A. calcoaceticus* (TA); and thiram spraying and inoculation with *R. intraradices* and *A. calcoaceticus* (TRA). Values are means ± standard error with three replicates. Analysis of variance (ANOVA) and Duncan’s test (honestly significant difference, HSD) were applied to evaluate significant differences (*P* < 0.05). Different lowercase letters indicate significant differences from different treatments (*P* < 0.05).
